# Mechanism-based myofilament manipulation to treat diastolic dysfunction in HFpEF

**DOI:** 10.3389/fphys.2024.1512550

**Published:** 2024-12-12

**Authors:** Katherine L. Dominic, Alexandra V. Schmidt, Henk Granzier, Kenneth S. Campbell, Julian E. Stelzer

**Affiliations:** ^1^ Department of Physiology and Biophysics, School of Medicine, Case Western Reserve University, Cleveland, OH, United States; ^2^ Department of Cellular and Molecular Medicine, University of Arizona, Tucson, AZ, United States; ^3^ Division of Cardiovascular Medicine, University of Kentucky, Lexington, KY, United States; ^4^ Department of Physiology, University of Kentucky, Lexington, KY, United States

**Keywords:** HFpEF, heart failure with preserved ejection fraction, cMyBP-C, cTnI, cardiac troponin I, titin, diastolic dysfunction, myosin binding protein C

## Abstract

Heart failure with preserved ejection fraction (HFpEF) is a major public health challenge, affecting millions worldwide and placing a significant burden on healthcare systems due to high hospitalization rates and limited treatment options. HFpEF is characterized by impaired cardiac relaxation, or diastolic dysfunction. However, there are no therapies that directly treat the primary feature of the disease. This is due in part to the complexity of normal diastolic function, and the challenge of isolating the mechanisms responsible for dysfunction in HFpEF. Without a clear understanding of the mechanisms driving diastolic dysfunction, progress in treatment development has been slow. In this review, we highlight three key areas of molecular dysregulation directly underlying impaired cardiac relaxation in HFpEF: altered calcium sensitivity in the troponin complex, impaired phosphorylation of myosin-binding protein C (cMyBP-C), and reduced titin compliance. We explore how targeting these pathways can restore normal relaxation, improve diastolic function, and potentially provide new therapeutic strategies for HFpEF treatment. Developing effective HFpEF therapies requires precision targeting to balance systolic and diastolic function, avoiding both upstream non-specificity and downstream rigidity. This review highlights three rational molecular targets with a strong mechanistic basis and potential for therapeutic success.

## Introduction

Heart failure (HF) is a clinical syndrome wherein the heart is unable to execute its primary function of efficiently circulating blood throughout the body in keeping with physiological demands (Zipes et al., 2019). HF is broadly characterized according to the percent of the ventricular volume displaced by each contraction, known as the ejection fraction (EF). In HF with reduced ejection fraction (HFrEF), the primary deficit is the reduced ability of the pump to propel blood forward during systole. HF with preserved EF (HFpEF), meanwhile, is mediated by impaired extensibility and relaxation of the heart muscle during diastole which leads to decreased ventricular filling and elevated filling pressure (Borlaug and Paulus, 2011; Reddy and Borlaug, 2016; Sharma and Kass, 2014).

Systolic and diastolic function are interrelated, and as such, EF-based classification is an oversimplification of the underlying pathophysiology (Borlaug and Paulus, 2011; Mishra and Kass, 2021). In both HFrEF and HFpEF, the clinical syndrome results from insufficient cardiac output, which includes fluid buildup in the lungs leading to impaired oxygenation, chronic activation of the renin-angiotensin-aldosterone system, and lack of cardiac reserve that results in exercise intolerance (Borlaug and Paulus, 2011; Sharma and Kass, 2014; Mishra and Kass, 2021; Paulus et al., 2007). These symptoms of impaired pump function exacerbate one another in a vicious cycle, ultimately reducing quality of life and significantly increasing morbidity and mortality (Reddy and Borlaug, 2016; Martin et al., 2024).

HF poses a massive global public health problem, impacting over 64 million people worldwide (Lippi and Sanchis-Gomar, 2020). In the US alone, 6.7 million adults have HF, projected to increase to over 8 million by 2030, accounting for 3% of the total population (Martin et al., 2024; Bozkurt et al., 2024). Approximately half of HF patients are diagnosed with either HFrEF or HFpEF, with the proportion of HFpEF patients projected to increase due to rising rates of HFpEF risk factors like diabetes, obesity, and aging of the population (Dunlay et al., 2017; Tsao et al., 2018). While several therapies exist for HFrEF that demonstrate mortality benefit, the effectiveness of these therapies decreases with increasing EF (Martin et al., 2024), such that there are very few evidence-based options available for treating HFpEF (Heidenreich et al., 2022). At the core of HF is the heart’s inability to meet the body’s physiological demands for blood flow. Addressing this requires either increasing the heart’s functional capacity or reducing its workload. The few therapies available for HFpEF do not directly address the underlying diastolic dysfunction that limits the capacity of the heart (Redfield, 2016; Janssen, 2019). Current first-line treatment for HFpEF is use of SGLT2 inhibitors, whose primary effect is on the kidney rather than the heart (Vaduganathan et al., 2022; Solomon et al., 2022; Anker et al., 2021). While SGLT2 inhibitors were shown to decrease the risk of HF-associated hospitalization, they have not shown a mortality benefit versus placebo (Vaduganathan et al., 2022; Solomon et al., 2022; Anker et al., 2021). Furthermore, the mechanism behind their cardiovascular benefit is unknown. The current management strategy for HFpEF patients involves attempting to mitigate comorbidities, for example by using GLP-1 agonist medications to treat underlying obesity that may have precipitated HFpEF (Anker et al., 2021; Ostrominski et al., 2024; Kosiborod et al., 2024; Kosiborod et al., 2023). Otherwise, current therapies such as diuretics, mineralocorticoid receptor antagonists, and combination angiotensin receptor/neprilysin inhibitors serve only to mitigate the symptoms of dysfunction by reducing the demand on the heart (Heidenreich et al., 2022). A mechanism-based approach that addresses the root cause of dysfunction, i.e. impaired filling, therefore remains an urgent yet unmet clinical need.

## Determinants of cardiac function

**FIGURE 1:**
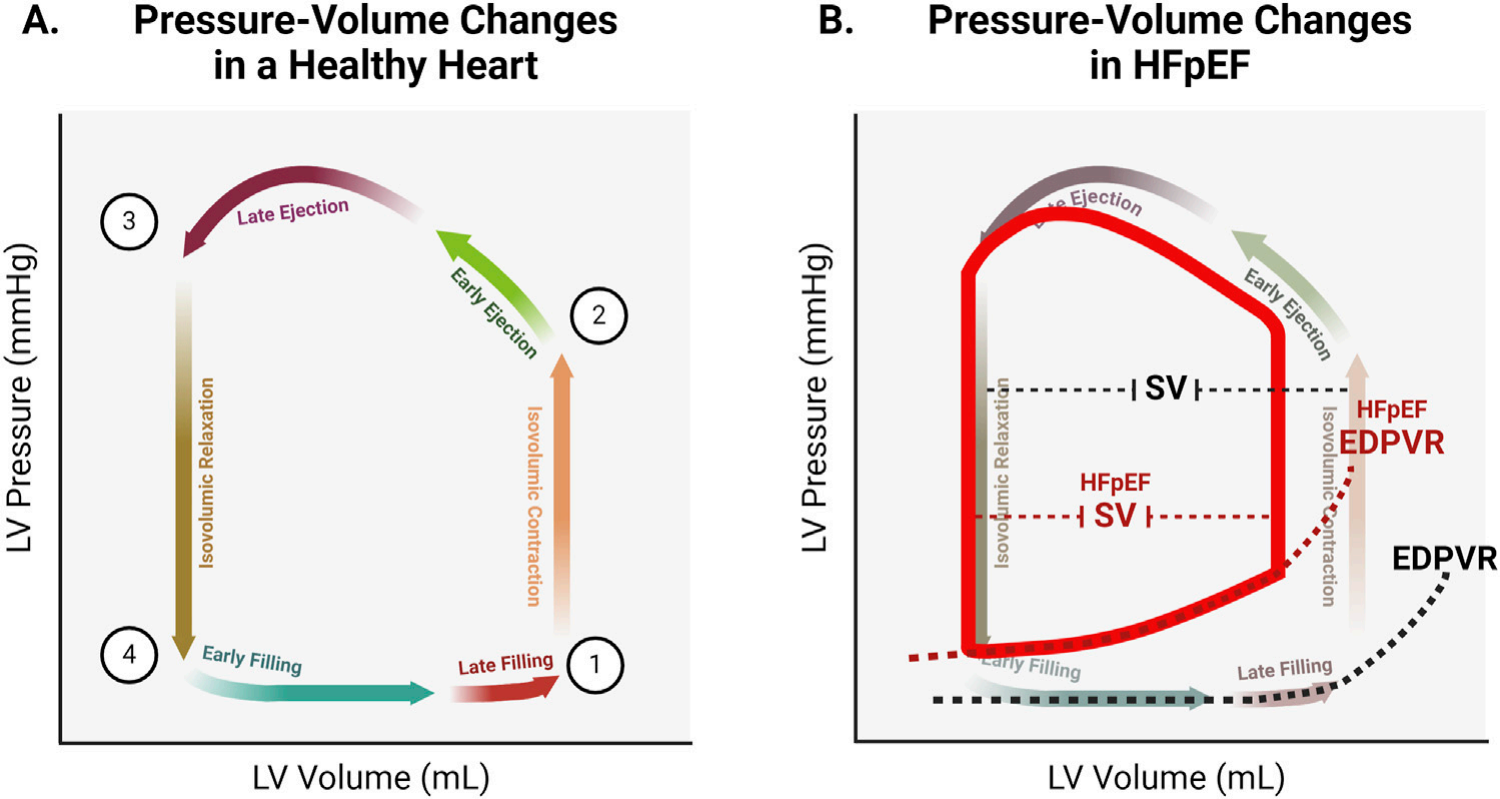
**(A)**. Representative pressure-volume changes in a healthy heart. At (Zipes et al., 2019), both the mitral and aortic valves are closed. At point (Borlaug and Paulus, 2011), the aortic valve opens. At point (Reddy and Borlaug, 2016), the aortic valve closes. At point (Sharma and Kass, 2014), the mitral valve opens. **(B)**. Representative changes to the pressure-volume relationship in HFpEF. Increased chamber stiffness results in increased steepness of the end diastolic pressure volume relation (EDPVR). Pressure within the stiffer ventricle is higher (upward shift of the pressure-volume loop). Elevated filling pressure results in decreased filling volume (left shift of the pressure-volume loop). Decreased filling volume leaves less volume for ejection, resulting in a decreased stroke volume (SV) and therefore decreased cardiac output.

Understanding the determinants of cardiac relaxation is essential for identifying therapeutic targets to restore diastolic function. The pressure-volume relation of the cardiac cycle described below is reviewed in detail in textbooks by Katz (Katz, 2011) and Klabunde (Klabunde, 2022). Briefly, each cardiac cycle begins following an electrical impulse that reaches the myocardium and initiates pressure development. At this stage, pressure in the left ventricle (LV) is above that in the left atrium (LA), so the mitral valve is closed, yet remains below the pressure in the aorta, so the aortic valve is also closed (Figure 1A, position 1). The ventricle undergoes isovolumic contraction as force generation against the closed valves causes pressure in the LV to rise. When pressure exceeds that in the aorta, the aortic valve opens and the forceful contraction of the LV drives ejection (Figure 1A, position 2). During early ejection, pressure continues to rise even as the volume of blood in the LV decreases. LV pressure reaches its peak during late ejection, then begins to fall as the volume in the LV reaches its nadir (known as end systolic volume, or ESV; Figure 1A position 3). Once pressure falls below that in the aorta, the aortic valve closes and the LV relaxes isovolumically. This isovolumic relaxation causes pressure to fall, eventually falling below the pressure in the LA. This pressure gradient results in the opening of the mitral valve and the flow of blood from LA to LV (Figure 1A, position 4). Pressure remains low during early filling as the recoil and untwisting of the contracted LV sucks blood into the LV (Yellin et al., 1990; Ashikaga et al., 2004), and the compliance of the LV wall partially resists pressure increase with passive stretch. During late filling, pressure in the LV begins to rise as the momentum of the LV muscle forcefully re-lengthening wanes, and the wall stretches further beyond its resting length. At the end of filling, LA contraction provides a final push of blood into the LV. At this point, rising ventricular pressure matches atrial pressure, leading to closure of the mitral valve, and returning the LV to the isovolumic state prior to the next contraction. The same events occur in the right atrium and ventricle, though at much lower pressures.

**FIGURE 2:**
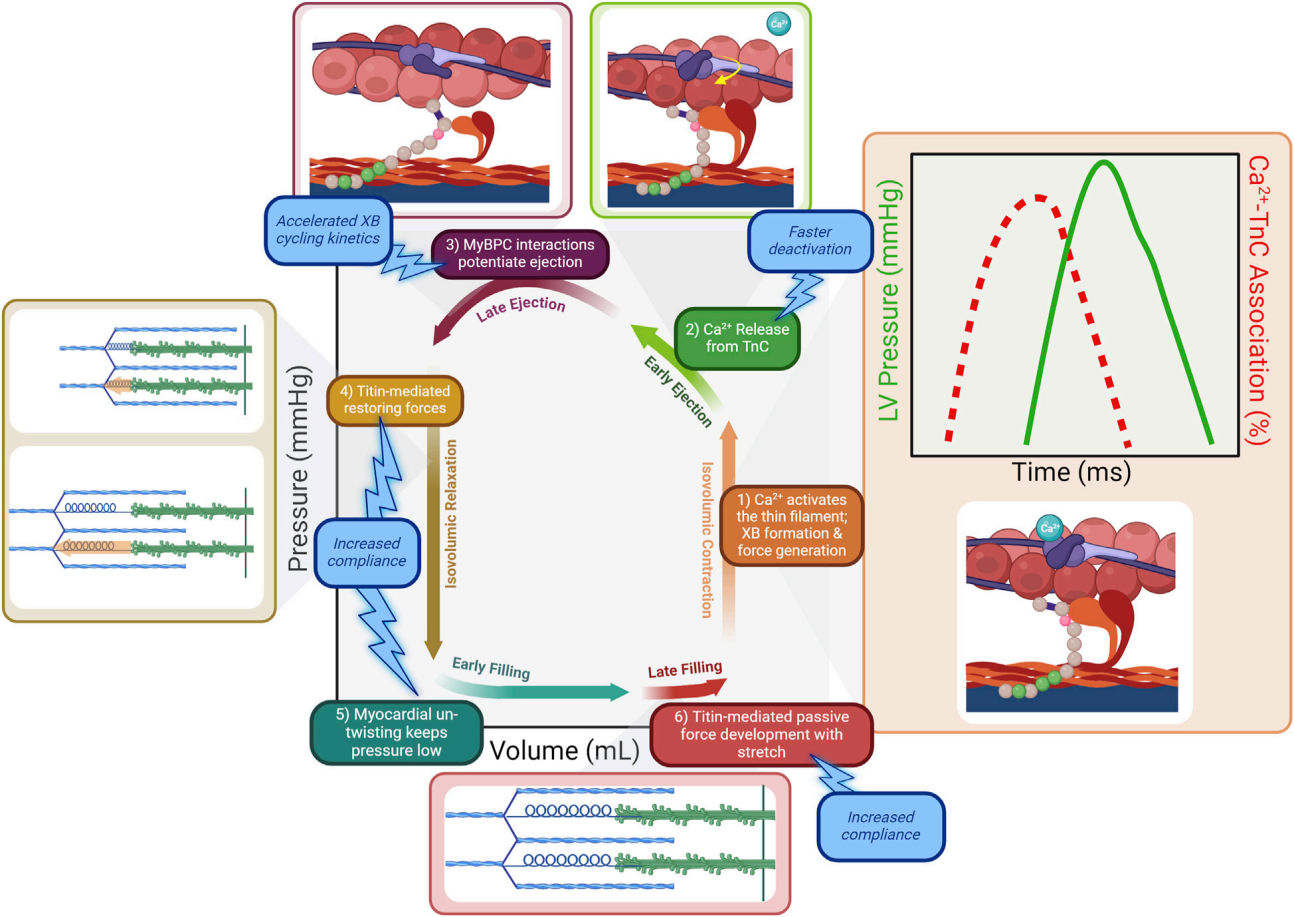
The molecular determinants within the sarcomere underlying the events of the cardiac cycle. 1) Following calcium release from the sarcoplasmic reticulum, calcium binds to cTnC and activates the thin filament. Cross-bridges (XBs) strongly bind the activated thin filament and pressure rises as cross-bridges enter the force-bearing state. The calcium transient peaks and declines prior to the peak of LV pressure generation (inset). 2) As the calcium transient declines during early ejection, calcium dissociates from cTnC. Targeting cTnC to promote faster calcium dissociation would result in faster deactivation of the thin filament, counteracting the prolonged activation in HFpEF. 3) LV pressure development continues as cMyBP-C potentiates the force-bearing state of cross-bridges. Therapeutically targeting cMyBP-C to accelerate XB cycling kinetics would counteract the prolonged maintenance of force development in HFpEF and allow faster relaxation. 4) Compression on titin springs is relieved as contraction ends, and the restoring force of titin re-lengthening facilitates relaxation. 5) Myocardial un-twisting creates a suction force to draw blood into the low-pressure ventricle. 6) Pressure develops during late filling due to passive stretch of titin. Promoting more compliant titin would facilitate improved relaxation by resisting pressure development as the ventricle relaxes and stretches during filling.

The organ-level events in the cardiac cycle are driven by corresponding molecular events within cardiomyocytes. Excitation-contraction coupling (ECC) is the process by which an electrical event initiates the contractile action of a muscle cell. Contraction is initiated by a sharp increase in intracellular calcium concentration, known as the calcium transient (Figure 2; box 1) (Bers, 2002; Biesiadecki et al., 2014). This correlates with the arrival of the electrical impulse. Voltage-gated L-type calcium channels open to allow entry of a small amount of calcium, which serves as the stimulus for a much greater release of stored calcium from the sarcoplasmic reticulum in a process known as calcium-induced calcium release (CICR). Calcium strongly activates the thin filament within sarcomeres. Prior to calcium release, a population of myosin cross-bridges exist in a weakly bound state, primed for strong binding contingent upon exposure of actin binding sites (Hinken and Solaro, 2007; Brunello and Fusi, 2024). At normal diastolic calcium levels, these actin binding sites are blocked by tropomyosin, under the regulation of the troponin complex consisting of cardiac troponin C (cTnC), troponin I (cTnI), and troponin T (cTnT) (Kobayashi and Solaro, 2005; Tardiff, 2011; Davis and Tikunova, 2008). When calcium levels rise at the start of systole, binding of calcium to cTnC promotes the activated state of the thin filament (Yamada et al., 2020; Risi et al., 2024). In the activated state, cTnI binds cTnC and stabilizes cTnC-calcium binding, while cTnT shifts tropomyosin to expose the myosin binding sites on actin (Biesiadecki et al., 2014). As long as the thin filament is in this activated state, primed myosin cross-bridges will strongly bind with actin and generate force, causing cardiac muscle to contract (Hinken and Solaro, 2007; Brunello and Fusi, 2024). The amount of calcium released during CICR as well as the binding kinetics and affinity of cTnC for calcium help control the magnitude and rate of thin filament activation, and thus the magnitude and rate of force generation in early systole.

The activation level of the thick filament and priming of the thick filament just prior to calcium release also contribute to the rate of early pressure development and shortening (Hinken and Solaro, 2007; Brunello and Fusi, 2024). The level of activation of the thick filament is regulated in part by a thick-filament associated protein cardiac myosin binding protein C (cMyBP-C) (Pfuhl and Gautel, 2012; Heling et al., 2020; Suay-Corredera and Alegre-Cebollada, 2022). When the phosphorylation level of cMyBP-C is low, cMyBP-C promotes the OFF state of the thick filament with myosin heads tethered to the thick filament backbone, reducing the population that exist in the weakly bound state that are primed for binding (Zoghbi et al., 2004; Al-Khayat et al., 2013; Zoghbi et al., 2008). When phosphorylation is high, cMyBP-C relieves this inhibition and promotes the ON state of the thick filament in which myosin heads project away from the thick filament backbone and are primed for interaction with the actin filament (Kensler et al., 2017; Ponnam et al., 2019; Rahmanseresht et al., 2021). When calcium levels are low just prior to the initiation of a new contraction, cMyBP-C also primes the thin filament for activation through its interactions with actin, sensitizing the thin filament to myosin binding (Kampourakis et al., 2014; Mun et al., 2014; Previs et al., 2016).

The calcium transient, as its name implies, exists only for a short period of time. Calcium is quickly removed from the cytosol by reuptake into the sarcoplasmic reticulum via the ATPase SERCA2a and extrusion from the cell via the sodium-calcium exchanger NCX1. These and other calcium handling processes are reviewed in detail elsewhere (Bers, 2002; Biesiadecki et al., 2014). Importantly, peak cytosolic calcium concentration occurs well before peak ventricular pressure is attained, at which point calcium binding has waned to nearly diastolic levels (Figure 2) (Biesiadecki et al., 2014; Hinken and Solaro, 2007; Monasky et al., 2008). Thus, late ejection is attributable to intrinsic sarcomere processes that potentiate force generation. Cooperative mechanisms within the sarcomere maintain thin filament activation beyond the decline of the calcium transient. Strongly bound cross-bridges increase the affinity of cTnC for calcium, prolonging activation (Gordon and Ridgway, 1993; Pan and Solaro, 1987; Landesberg, 1996). Additionally, strongly bound cross-bridges act as a “foot in the door” of the thin filament regulatory unit, and sterically block tropomyosin from covering adjacent myosin binding sites on actin (Gordon et al., 2000; Moss et al., 2004; Tobacman, 1996). Perhaps the most important mechanism by which ejection is prolonged is the activity of cMyBP-C. Its importance is underlined by studies demonstrating that ejection time is severely truncated when cMyBP-C is absent from sarcomeres (Harris et al., 2002; Tong et al., 2008; Mamidi et al., 2014; Stelzer et al., 2006a). As mentioned previously in the context of priming the sarcomere prior to contraction, cMyBP-C again contributes to the continued activation of the thin filament as calcium levels wane during late systole (Heling et al., 2020; Kampourakis et al., 2014; Mun et al., 2014; Previs et al., 2016). Its interactions with the thin filament also create a viscous drag that slows shortening, reducing shortening-induced deactivation (Walcott et al., 2015; Weith A. et al., 2012). Overall, cMyBP-C acts as a brake on the system, slowing and prolonging the force generating state of the sarcomere (Figure 2; box 3).

Late ejection is terminated as sarcomere shortening induces strain on active cross-bridges, resulting in shortening-induced deactivation (Martyn et al., 1983; McDonald and Moss, 2000). This force ultimately overpowers the dissipating cooperative mechanisms that maintain activation of the sarcomere, and strongly-bound cross-bridges detach (Hinken and Solaro, 2007). Once detachment occurs, re-binding is prevented in the absence of calcium, as the thin filament’s regulatory unit returns to its position blocking the myosin binding sites on actin.

As the forces favoring shortening of the sarcomere decline, the forces favoring re-lengthening take over. One such force at the level of the sarcomere is the potential energy stored during contraction by the compression of the sarcomere protein titin (Granzier et al., 2002; Linke, 2023). Titin is a giant protein that spans the thick filament from Z-disk to M-band and serves as a myofibril scaffold for the correct positioning and stoichiometry of other thick filament proteins (LeWinter and Granzier, 2014; Tonino et al., 2017). In addition, titin’s extensible I-band segment functions as a molecular spring that contributes to the rate of cardiac relaxation (Granzier et al., 2002). Titin’s extensible I-band does not begin exactly at the edge of the Z-disk. Instead, a short segment of titin adjacent to the Z-disk binds to the thin filament, making it stiff and inextensible (Helmes et al., 1996). This structure enables titin’s extensible region to act as a bi-directional spring. When sarcomeres are stretched beyond their slack length, titin generates a force that pulls the Z-disks toward each other. Conversely, when sarcomeres shorten below the slack length, titin exerts a force that pushes the Z-disks apart—this force is known as the restoring force.

The restoring force is engaged during systole when sarcomere shortening occurs below the equilibrium volume and the stored potential energy is expected to contribute to the suction force essential for the early filling phase of the heart (Bell et al., 2000) Thus, when contraction ends, the restoring force of titin re-lengthening drives relaxation (Figure 2; box 4). This forceful re-lengthening generates a force in the opposite direction from contraction, resulting in a drop in ventricular pressure as cardiac muscle relaxes. Elements outside the sarcomere also contribute to the restoring force following contraction. Compression of microtubules causes them to buckle, adopting a high-energy conformation that bears the compressive load of contraction (Robison et al., 2016; Caporizzo and Prosser, 2022).

Another force promoting negative pressure generation in the ventricle is the release of stored momentum from contraction in the form of ventricular untwisting (Figure 2; box 5) (Burns et al., 2009; Opdahl et al., 2012). Myofibrils in the epi- and endocardium are arranged in opposite directions to one another such that contraction results in twisting of the ventricle–a motion similar to wringing a towel (Sengupta et al., 2008; Omar et al., 2015). The momentum of this twist is reversed following contraction and ventricular untwisting contributes to ventricular suction during relaxation. As such, the force and velocity of shortening during systole are themselves important determinants of diastolic function.

Once pressure in the ventricle has fallen below that in the atrium, blood begins to fill the ventricle. As the volume in the ventricle increases, the elastance of the myocardium determines the corresponding change in pressure. Titin stiffness is the primary determinant of this passive pressure during filling (Granzier and Irving, 1995). Other elements such as the cytoskeleton (including microtubules, actin, and intermediate filaments) as well as the extracellular matrix (ECM) also contribute to stiffness. While titin alone is the primary determinant of elastic passive force, the cytoskeletal elements are relevant to the viscous component (Loescher et al., 2023; Loescher and Linke, 2024). Pressure remains low during early filling as titin’s early high compliance allows for large changes in volume with correspondingly small changes in pressure (Linke, 2023; Helmes et al., 1999). As ventricular volume reaches the threshold of titin’s compliance, titin is the primary determinant of muscle stiffness (Loescher and Linke, 2024; Chung and Granzier, 2011). Thus during late filling (Figure 2; box 6), ventricular pressure rises slightly with the rise in volume. This relationship is characterized by the slope of the end diastolic pressure-volume relation, or EDPVR (Figure 1B).

The stretch of the ventricle experienced at end diastole is a primary determinant of muscle contractility in the subsequent cardiac cycle, according to the well-described Frank-Starling mechanism (Konhilas et al., 2002). Thus diastolic performance, and the volume of blood that is able to enter the ventricle during filling (end diastolic volume, or EDV), is itself an important determinant of systolic function–a reciprocal of the relationship between systolic performance and diastolic performance. This exemplifies the cyclic and interrelated nature of cardiac function. The pressure gradient that exists between atrium and ventricle is the driving force behind ventricular filling. The larger the pressure gradient, the greater the driving force. The greater the driving force, the faster the flow, and thus greater the volume of blood that can enter the ventricle before the pressure equalizes and filling ceases due to closure of the valve. By keeping pressure in the ventricle low, and even generating negative pressure during early relaxation, titin’s compliant, spring-like qualities allow for efficient filling of the ventricle without requiring elevated atrial pressure (Linke, 2023).

## Dysfunction in HFpEF

The organ-level diastolic dysfunction that underlies HFpEF is attributable to dysfunction of the different sarcomere processes that drive normal relaxation at baseline and in response to stress (Mishra and Kass, 2021; Knight and Woulfe, 2022; van der Velden and Stienen, 2019; Aboonabi and McCauley, 2024). HFpEF can develop in the context of a variety of different whole-body perturbations, including diabetes, obesity, and aging (Borlaug and Paulus, 2011; Reddy and Borlaug, 2016; Paulus and Tschöpe, 2013). What these different conditions have in common is ultimately dysfunction of cardiac mechanics that become limiting. Potential culprits underlying this mechanical dysfunction include signaling pathways involving different kinases and growth factors. Recently, modulation of receptor tyrosine kinase signaling was found to promote cardiac relaxation (Algül et al., 2023). The PKA/PKG signaling axis in particular is known to be dysregulated in the setting of HF (Mishra and Kass, 2021; Paulus and Tschöpe, 2013; Hamdani et al., 2013a). Targets of this axis include proteins described above that regulate the cardiac cycle, including cTnI, cMyBP-C, and titin.

Under physiologic conditions, activation of PKA signaling by adrenergic stress results in cTnI phosphorylation (Kranias and Solaro, 1982; Kentish et al., 2001; Peña and Wolska, 2004; Kooij et al., 2010; Biesiadecki et al., 2007). Phosphorylation of cTnI decreases the calcium sensitivity of cTnC (Kentish et al., 2001). In the face of the increased calcium release that simultaneously occurs during adrenergic stress, the rate of force development still increases, but the decreased sensitivity helps prevent over-activation of the thin filament and tunes de-activation to match the overall faster rate of contraction (Bers, 2002). In HFpEF, cTnI phosphorylation is decreased (Hamdani et al., 2013a), so cTnC has a higher calcium sensitivity than required for the contractile state. This leads to prolonged activation that encroaches on the time needed for pressure to fully drop during diastole.

cMyBP-C phosphorylation by PKA decreases its affinity for both actin and myosin (Pfuhl and Gautel, 2012; Kensler et al., 2017; Ponnam et al., 2019; Tong et al., 2008). While unphosphorylated cMyBP-C acts as a brake on the system, constraining and slowing force generation, phosphorylation of cMyBP-C is like stepping on the gas (Previs et al., 2016; Gresham and Stelzer, 2016; Weith AE. et al., 2012). Decreased interaction between phosphorylated cMyBP-C and myosin relieves inhibition on myosin heads, resulting in greater activation of the thick filament and more cross-bridges added to the force generating pool (Kensler et al., 2017; Ponnam et al., 2019). While decreased interaction between cMyBP-C and actin diminishes the activating effect on the thin filament, this effect is dwarfed by the strongly activating effect of increased calcium release during normal adrenergic signaling (Bers, 2002). Decreased interaction between cMyBP-C and actin thus has a net accelerating effect because of the reduction in viscous drag that is otherwise imposed when cMyBP-C links the thick and thin filaments (Walcott et al., 2015). PKA phosphorylation of cMyBP-C has been shown to accelerate overall cross-bridge cycling kinetics (Weith A. et al., 2012; Gresham and Stelzer, 2016; Weith AE. et al., 2012; Stelzer et al., 2006b). Because cMyBP-C contributes to the potentiation of force generation during late systole, acceleration of cycling kinetics results in shortened ejection (Gresham and Stelzer, 2016). This is necessary for tuning the mechanics of contraction and relaxation to fit the externally fixed time between heartbeats. In HFpEF, cMyBP-C phosphorylation is decreased (Hamdani et al., 2013a), and the normal acceleration of contraction kinetics in response to stress is blunted. Ejection is inappropriately prolonged, leaving inadequate time for relaxation. Additionally, the slower contractile kinetics may limit the extent and momentum of myocardial twisting during systole that is necessary for optimal re-lengthening during diastole.

Titin is also a target for a variety of signaling pathways, including PKA and PKG (Hamdani et al., 2013a; Borbély et al., 2009; Hamdani et al., 2013b; Krüger et al., 2009; Yamasaki et al., 2002). During adrenergic stress, phosphorylation of titin increases its compliance, limiting the passive force developed during filling (Hamdani et al., 2013b; Krüger et al., 2009; Yamasaki et al., 2002). Additionally, different titin isoforms exhibit different inherent stiffness (Granzier et al., 2000), and isoform expression is regulated by signaling pathways that are perturbed in HFpEF (Hamdani et al., 2013a). In HFpEF, titin phosphorylation is decreased, and expression of the stiffer isoform increases (Hamdani et al., 2013a; Zile et al., 2015). These factors contribute to the increased overall myocardial stiffness observed in HFpEF hearts, and help explain the increased slope of the EDVPR (Figure 1B). While titin is the primary determinant of passive stiffness in the heart, other cellular and extracellular elements also contribute and are the determinants of the viscous component (Loescher et al., 2023). Increased ventricle stiffness in HFpEF is also the result of excess collagen deposition in the ECM and increased cardiac fibrosis (Zile et al., 2015). Further, microtubule expression and tyrosination is altered in HFpEF, leading to increased LV stiffness (Schulz et al., 2022; Caporizzo et al., 2020).

The decreased diastolic filling that occurs due to altered contractile kinetics, slowed relaxation, and increased stiffness results in limitation of stroke volume (Figure 1B) (Klabunde, 2022). Stroke volume represents the blood that is supplied to the body with each heartbeat, and must supply the metabolic demands of all tissues. A stiffer heart with a slowed rate of relaxation and less total time for relaxation will fill with less blood. In order to fill a stiffer heart with an elevated ventricular pressure during filling, a correspondingly higher atrial pressure is required to maintain the gradient to drive filling. Decreases in stroke volume are the ultimate source of cardiac insufficiency in HFpEF, and elevations in left atrial and pulmonary vascular pressure directly and indirectly result in comorbidities such as atrial fibrillation and pulmonary hypertension (Zakeri et al., 2013; Lam et al., 2009; Reddy and Borlaug, 2021). Therapeutic interventions should therefore target the factors that result in decreased stroke volume and elevated filling pressure in order to ameliorate this insufficiency and restore cardiac function. This highlights cTnI, cMyBP-C, and titin as potentially ideal targets.

## Therapeutic outlook

The therapeutic strategies currently employed in HFpEF are only tangentially aimed at addressing the underlying myofilament dysfunction that is at the root of the syndrome. Attempts have been made to target the dysregulated PKG signaling axis that is likely in part responsible for myofilament dysfunction, for example via phosphodiesterase inhibition (Redfield et al., 2013; Hoendermis et al., 2015). However, this target lies far upstream in the signaling pathway and thus lacks specificity, which likely explains its lack of clinical success. Attempts to target the PKA signaling pathway via beta-blockers have similarly been unsuccessful in HFpEF (Cleland et al., 2018). While SGLT2 inhibitors have shown initial promise in HFpEF, the mechanism underlying their cardiac effects remains unclear. Given that SGLT2 is not significantly expressed in the myocardium, it seems likely that the effect is systemic or off-target, rather than targeted to the sarcomere (Dyck et al., 2022; Cowie and Fisher, 2020; Lopaschuk and Verma, 2020). At present, there are no sarcomere-based therapies approved for use in HFpEF. The myosin-inhibiting small molecule mavacamten has shown promise in a mouse model of HFpEF (Lin et al., 2022). However, targeting myosin to address cardiac dysfunction has proven challenging in the past, for example with the development of novel inotropes aimed at improving function in HFrEF such as omecamtiv mecarbil (Teerlink et al., 2011; Lewis et al., 2022; Mamidi et al., 2017). The drawback of targeting myosin itself is that physiologically, cardiac myosin is required to perform two contradictory functions. Drugs that directly promote the contractile function of myosin do so at the expense of relaxation. On the other hand, drugs that inhibit contraction in order to promote relaxation induce systolic dysfunction. More promising are examples of therapeutic approaches that target proteins that are known to be altered in HFpEF whose native function involves tuning the sarcomere to appropriately match systolic and diastolic function. These targets include the proteins described in this review: the troponin complex, cMyBP-C, and titin.

Given that one of the primary molecular defects in HFpEF is inappropriately increased calcium sensitivity of the sarcomere as a result of impaired cTnI phosphorylation, reducing calcium sensitivity is a therapeutic goal. Attempts to target the troponin complex have involved methods that decrease calcium sensitivity to reverse this dysfunction. The small molecule W7 decreases the calcium sensitivity of force development in cardiac muscle by decreasing the binding of cTnI to cTnC (Cai et al., 2018). This agent has been tested in pre-clinical disease models characterized by known hyper calcium-sensitive sarcomere states, such as hypertrophic and restrictive cardiomyopathy (Thompson et al., 2016). Novel protein engineering techniques have also been investigated to alter the calcium sensitivity of the thin filament. In a model of decreased contractility induced by myocardial infarction, using AAV9-based gene therapy to deliver cTnC with a calcium-sensitizing mutation therapeutically enhanced cardiac function (Davis et al., 2016; Shettigar et al., 2016). The success of this approach in a HFrEF model suggests that it could be further developed to apply to models of HFpEF by engineering mutations that counteract the increased calcium sensitivity that contributes to HFpEF pathophysiology. Whatever the approach, targeting the troponin complex would be expected to mitigate diastolic dysfunction by shortening early ejection and promoting faster deactivation of the thin filament, resulting in improved coupling of systolic and diastolic performance (Figure 2; box 2).

Decreased phosphorylation of cMyBP-C in HFpEF results in slowed cross-bridge cycling kinetics, decreased myosin head recruitment, and increased viscous drag (Kensler et al., 2017; Tong et al., 2008; Hamdani et al., 2013a; Gresham and Stelzer, 2016; Stelzer et al., 2006b). Therefore, therapies that either promote or mimic the phosphorylated state of cMyBP-C would be expected to restore function. Currently, there are no small molecules with cMyBP-C as their target. However, the recent development of a high-throughput screen for identifying compounds that alter cMyBP-C binding makes this a promising avenue for further investigation (Bunch et al., 2023; Dvornikov et al., 2023; Kanassatega et al., 2022; Wong et al., 2024). There are challenges inherent in the development of small molecules to target such subtle and pleiotropic effects as cMyBP-C phosphorylation. The advent of gene therapy provides an opportunity for more rational design of therapies by using protein engineering. Similar to the approach developed for delivering a mutated version of cTnC with altered calcium sensitivity, mutated versions of cMyBP-C that structurally and functionally mimic the phosphorylated state could be used to tune the sarcomere. The result of therapies that successfully target cMyBP-C would be acceleration of shortening velocity, shortening of late ejection, and enhancement of cooperative deactivation. Accelerating shortening velocity harnesses the mechanical coupling between systolic and diastolic function to improve relaxation by providing more stored momentum during systole that will be released in diastole. Overall acceleration of kinetics and enhancement of cooperative deactivation shortens the duration of ejection, providing more time during a given cardiac cycle for adequate relaxation and filling (Figure 2; box 3).

Decreased titin compliance is one of the primary sarcomeric factors underlying diastolic dysfunction in HFpEF (Tamargo et al., 2023). Stiffer titin results in a steeper EDVPR, which contributes to decreased EDV as described above. Increased titin stiffness in HFpEF is the result of alterations in phosphorylation and titin isoform expression (Hamdani et al., 2013a; Zile et al., 2015; Tamargo et al., 2023; Hamdani et al., 2013c). Studies in rat cardiomyocyte cultures indicate that the glucose-lowering drug metformin and the growth factor neuregulin-1 enhance ERK1/2 activity, leading to increased phosphorylation at multiple titin sites (Hopf et al., 2018). The potential therapeutic benefits of metformin were explored in a mouse model with HFpEF-like symptoms (Slater et al., 2018). In these metformin-treated mice, phosphorylation of titin was increased. Although metformin did not affect extracellular matrix stiffness, it reduced titin-based passive stiffness, normalized left ventricular diastolic dysfunction, and improved exercise tolerance (Slater et al., 2018). Therapeutic strategies aimed at restoring titin compliance by promoting expression of more compliant isoforms have also been successful in preclinical animal models of HFpEF (Methawasin et al., 2016; Radke et al., 2021). The splicing factor RBM20 has been shown to mediate shifts to stiffer titin isoforms, and blocking the function of this splicing factor in transgenic animals resulted in improved compliance (Methawasin et al., 2016). In a mouse model of HFpEF and in human engineered heart tissue, using antisense oligonucleotides to inhibit RBM20 resulted in increased expression of compliant titin isoforms and improvements in *in vivo* and *in vitro* measures of diastolic function (Radke et al., 2021). The success of this approach illustrates the principal role that titin alterations play in HFpEF pathophysiology, and demonstrates that targeting titin to directly address this pathophysiology is beneficial. By increasing titin compliance in HFpEF hearts, the early phase of relaxation is accelerated (Figure 2; boxes 4 and 5), filling occurs at a lower pressure (Figure 2; box 6), and the slope of the EDVPR is normalized (Figure 1B).

## Conclusion

Normal cardiac diastolic function involves a high degree of complexity and requires precise interaction in time and space between countless moving parts, resulting in emergent phenomena that cannot be understood in isolation (Janssen, 2019). Added to this is the highly complex physiological milieu of comorbidities in which HFpEF develops. It is therefore not surprising that the challenge of effectively treating HFpEF remains unsolved. Such a complex process requires highly precise targeting. Too far upstream, as in the case of agents aimed at whole signaling pathways, and the target is too nonspecific with unintended off target effects that may ultimately exacerbate dysfunction. Too far downstream, as in the case of direct myosin modulators, and the target lacks the flexibility to encompass the multiple different modes of activity required for proper coupling of systolic and diastolic function. The targets proposed in this review represent promising intermediates, and should be the focus of therapy development with the best chance of success.
